# Effect of Immune Inducers on *Nosema ceranae* Multiplication and Their Impact on Honey Bee (*Apis mellifera* L.) Survivorship and Behaviors

**DOI:** 10.3390/insects11090572

**Published:** 2020-08-26

**Authors:** Pegah Valizadeh, Ernesto Guzman-Novoa, Paul H. Goodwin

**Affiliations:** 1 School of Environmental Sciences, University of Guelph, 50 Stone Road East, Guelph, ON N1G2W1, Canada; valizadehpegah@gmail.com (P.V.); pgoodwin@uoguelph.ca (P.H.G.); 2 Agricultural Research Education and Extension Organization of Iran, Animal Science Research Institute of Iran, Honey Bee Research Department, Shahid-Beheshty Ave., 1st Dehghan-villa, Karaj 3146618361, Iran

**Keywords:** *Nosema cerana*, *Apis mellifera*, foraging behavior, hygienic behavior, proboscis extension reflex, immune inducers, CCD

## Abstract

**Simple Summary:**

Nosema disease of honey bees is caused by the fungus *Nosema ceranae*, which multiplies and damages cells lining the digestive tract, impairing food digestion and debilitating the bees. Current control involves using antibiotics, which is undesirable because of possible antibiotic resistance of the fungus and contamination of honey. In this study, the natural compounds flagellin, zymosan, chitosan and peptidoglycan were investigated as alternatives for controlling *Nosema ceranae* infections and for their effect on bee survivorship and behaviors. Chitosan and peptidoglycan reduced infection and increased survivorship of infected bees. However, neither compound altered the bees’ hygienic behavior, which was also not affected by the infection. Chitosan treated bees collected more pollen and nectar than healthy and infected bees. Memory in the bees was temporarily impaired by chitosan but was not affected by peptidoglycan, nor was it affected by *Nosema ceranae*. This study shows that chitosan and peptidoglycan provide benefits by partially reducing *Nosema ceranae* infection while increasing survivorship of honey bees. Also, chitosan and peptidoglycan increased the collection of pollen and nectar, which may improve bee health and colony productivity. These benefits could result in more honey produced, more crops pollinated and more healthy bee colonies.

**Abstract:**

Nosema disease is a major disease of honey bees caused by two species of microsporidia, *Nosema apis* and *N. ceranae*. Current control involves using antibiotics, which is undesirable because of possible antibiotic resistance and contamination. In this study, flagellin, zymosan, chitosan, and peptidoglycan were investigated as alternatives for controlling *N. ceranae* infections and for their effect on bee survivorship and behaviors. Chitosan and peptidoglycan significantly reduced the infection, and significantly increased survivorship of infected bees, with chitosan being more effective. However, neither compound altered the bees’ hygienic behavior, which was also not affected by the infection. Chitosan significantly increased pollen foraging and both compounds significantly increased non-pollen foraging compared to healthy and infected bees. Memory retention, evaluated with the proboscis extension reflex assay, was temporarily impaired by chitosan but was not affected by peptidoglycan, nor was it affected by *N. ceranae* infection compared to the non-infected bees. This study indicates that chitosan and peptidoglycan provide benefits by partially reducing *N. ceranae* spore numbers while increasing survivorship compared to *N. ceranae* infected bees. Also, chitosan and peptidoglycan improved aspects of foraging behavior even more than in healthy bees, showing that they may act as stimulators of important honey bee behaviors.

## 1. Introduction

*Nosema ceranae* and *N. apis* are two microsporidian fungi that cause nosema disease, an infection of the midgut of adult honey bees (*Apis mellifera*) [1]. *Nosema* spores enter the digestive tract following ingestion during food exchange or removal of contaminated fecal material, and then germinate invading epithelial cells of the bee’s midgut, where the parasite multiplies [2,3]. *N. ceranae* may shorten the life span of infected bees [4] and might also be a factor contributing to colony collapse disorder (CCD) [5,6,7].

Nosema disease negatively affects honey bees by rupturing the epithelial cells of the midgut, impairing food digestion [8]. The result is an increase in mortality of infected bees. For example, Higes et al. [4] inoculated *A. mellifera* with 125,000 *N. ceranae* spores per bee, and mortality on day seven was 94.1% compared to only 5.9% in the non-inoculated control. 

The health of a honey bee colony relies on the foraging behavior of its workers for the collection of pollen, nectar, water, and resins [9]. *Nosema* infection has a negative effect on foraging behavior. Foragers infected with *N. apis* failed to return to their hive [10], and *N. ceranae* and *N. apis* infection decreased pollen gathering [11]. However, it is unknown if other bee behaviors are affected, such as hygienic behavior, where bees detect a diseased or dead larva or pupa, uncap the cell and remove the brood. This important behavior reduces the incidence of brood infections and *Varroa destructor* infestations in honey bee colonies [12].

Foraging and hygienic behaviors require learning and memory [13,14,15]. However, *N. ceranae* does not seem to affect learning based on the proboscis extension reflex (PER) assay [16]. PER is a test of memory and learning based on the extension of the proboscis as a reflex to stimulation associated with a food reward (unconditioned stimulus) after training the bees with conditioned stimuli, such as odors [17,18]. Learning and memory are measured by assessing the association between the odor with the sugar reward [19].

Currently, control of *Nosema* infections involves the use of fumagillin, an antibiotic produced by the fungus *Aspergillus fumigatus* [20]. Although it has been commercially used for more than 50 years, there are concerns that *Nosema* spp. are becoming resistant to it [20], and residues of the antibiotic can be detected in hive products, such as honey and wax [21]. An alternative would be to use natural compounds that stimulate the immune system of the bee to allow the insect to better resist infection. Immune inducers are substances that manipulate the immunity of a host by inducing resistance against pathogens [22]. Many immune inducers are types of pathogen-associated molecular patterns (PAMPs) that are recognized by pattern-recognition receptors (PRRs) in the host, triggering the innate immune response [23]. Some examples of PAMPs are flagellin, which is a component of the flagella of bacteria [24], peptidoglycan, which is a component of cell wall in bacteria [22], and zymosan and chitosan, which are derived from components of the cell wall of fungi [23]. PAMPs are able affect honey bee gene expression, such as peptidoglycan and chitosan that increased expression of the immune-related genes *hymenoptaecin* and *defensin 2*, as well as *blue cheese*, which is an autophagy-related gene, in *N. ceranae* inoculated adult honey bees [25]. Peptidoglycan also induced expression of *abaecin* and *defensin 1* in honey bees [22]. 

This study examined the use of flagellin, zymosan, chitosan, and peptidoglycan, fed to honey bees in sucrose syrup, to reduce the effects of infection by *N. ceranae*. It was hypothesized that those immune inducers can reduce infection intensity of the pathogen as well as improve survivorship, foraging behavior, hygienic behavior, and memory.

## 2. Materials and Methods

### 2.1. Experimental Bees and Nosema Ceranae Spores

Newly emerged bees of the Buckfast strain were obtained from colonies established at the University of Guelph’s Honey Bee Research Centre in Guelph, ON, Canada. Combs with emerging brood were placed inside screened cages in an incubator (32 °C and 60% RH) overnight. Emerged bees were transferred to a plastic container and starved for 2 h prior to treatment. To ensure that the newly emerged bees were *Nosema*-free, a microscopy diagnosis was performed using 25 randomly selected emerged bees [26]. *Nosema ceranae* spores were extracted from a pool of *N. ceranae*-infected foragers collected from highly infected colonies. For spore extraction, abdomens were dissected from 20 bees and macerated in a mortar and pestle using 1 mL sterile dH_2_O per abdomen. The macerate was strained through a honey filter (1000 holes/cm^2^, Better Bee Supplies, Cambridge, ON, Canada) and then centrifuged at 200× g (Eppendorf, Symphony^TM^, Model 2417R, West Chester, PA, USA) for 8 min [27]. The pellets containing spores were suspended in sterile dH_2_O, and the spore concentration adjusted to 10^4^ spores/µL using a haemocytometer as per Cantwell [26].

### 2.2. PAMPs

The PAMPs used in this study were zymosan (from *Saccharomyces cerevisiae*) (Sigma, St. Louis, MO, USA), peptidoglycan (from *Bacillus subtilis*) (Sigma), chitosan (DNP, Quebec City, QC, Canada), and flagellin (from *Bacillus thuringiensis*) (Sigma).

### 2.3. Treatment of Bees with PAMPs and N. ceranae

Newly-emerged bees were randomly assigned to treatments and individually marked on the thorax with different colors of enamel paint, according to treatment. Subsequently, the marked bees were individually force fed 10 µL of solutions using a micropipetter (2–20 µL, Eppendorf, Westbury, NY, USA) containing either 50% sucrose syrup (negative control), 50% sucrose syrup containing 10^4^ spores/µL of *N. ceranae* (positive control), or 50% sucrose syrup containing 10^4^ spores/µL of *N. ceranae* plus 0.125 µg/µL zymosan, 1.0 µg/µL peptidoglycan, 1.0 µg/µL chitosan, or 0.1 µg/µL flagellin. The PAMP doses were chosen based on the following studies. The peptidoglycan dose was from Evans and Lopez [22] who fed it in sugar syrup to induce innate immune related genes in *A. mellifera*, the zymosan dose was based on Tanji et al. [28], who fed it to flesh flies, *Sarcophaga peregrine*, in sugar syrup to induce innate immunity, the chitosan dose was from Hashimoto et al. [29], who injected chitosan in sterile dH_2_O to induce innate immune related genes in the eri-silkworm, *Samia cynthia ricini*, and the flagellin dose was from Samakovlis et al. [24], who added it to a cell line culture of *Drosophila melanogaster* to induce an immune related gene.

After feeding, each bee was transferred to a screened hoarding cage (10 × 15 × 15 cm) until 60 bees of the same treatment had been introduced. Each cage was provided with two gravity feeders, one containing sterile dH_2_O and the other 50% sucrose syrup for the bees to feed *ad libitum*. The cages of all treatments were kept in an incubator at 32 °C and 60% RH for 20 days post treatment (dpt). Each treatment was tested with three biological replications.

### 2.4. N. ceranae Infection Intensity

*N. ceranae* infections were determined by randomly collecting 10 bees from each cage at 10 or 20 dpt as described above. Two samples from each macerate were analyzed and the numbers of *N. ceranae* spores/bee were averaged. After this experiment, the two PAMPs that significantly restrained the proliferation of *N. ceranae* spores, were selected for PER, survivorship, and behavioral testing in observation hives.

### 2.5. Observation Hives

Three observation hives were installed to study survivorship, hygienic, and foraging behavior of the experimental bees. Each observation hive (47.0 × 4.1 × 96.5 cm) was placed in a separate windowless room and was connected to a runway ramp (65 × 10 × 2 cm), which in turn was connected to an opening to allow the bees to forage normally outside the building. The ramp was covered with glass to allow the observation of foraging behavior. The runway was designed so that outgoing bees were deterred by a barrier from leaving the hive via the return lane [30]. Four newly-drawn combs with about 4000 worker bees of variable ages and a queen unrelated to the experimental bees were installed into each hive. Bees were emerged and treated as above, except for the *N. ceranae* + zymosan and *N. ceranae* + flagellin treatments because these PAMPs were less able to reduce spore loads in the treated bees of the cage experiments. A total of 500 bees per each of the four treatments were introduced into each observation hive (2000 experimental bees). The bees of the four treatments were co-fostered in common hives, which is a way of controlling environmental influences and to better separate the effects of the different treatments [31]. For hygienic behavior observations, a small door (10 × 10 cm) was installed at the bottom of the hive over the plexiglass to facilitate introducing a comb section containing frozen brood at the bottom of the lower frame.

### 2.6. Survivorship

The survivorship of the experimental bees in the observation hives was determined as per Unger and Guzman-Novoa [32] by performing visual censuses of the bees of each treatment during early mornings when bees were not foraging. To determine acceptance, the first census was done 24 h after introducing the bees to each of the observation hives and then continued daily from day 8 to 31. The survivorship of the bees for each treatment was estimated by calculating the proportion of bees present in the hive per census day over the initial number of accepted bees.

### 2.7. Hygienic Behavior

Hygienic behavior was evaluated by introducing to the hive a 4 × 4 cm section of a comb containing freeze-killed capped brood. The comb of the lower frame of each observation hive had an open square area of 4 × 4 cm that had been cut out using a knife. This open area faced the 10 × 10 cm lower plexiglass door of the observation hive, through which the comb section containing the frozen brood was introduced and fixed to the comb using toothpicks.

Two hygienic tasks of the bees, cell uncapping and brood removal, were recorded during 4 h of observation per day and three repetitions of the assay were conducted during three days, when the bees were 12, 14, and 15 days old. The proportion of bees of each treatment participating in hygienic tasks were calculated by dividing the number of bees uncapping cells or removing brood over the number of bees remaining alive for each treatment during the days of observation.

### 2.8. Foraging Behavior 

Foraging behavior of the bees was measured for 2 h in the early morning and 2 h in late afternoon, each of three days per week on weeks 2–4 post-introduction to the observation hives. Foraging was assessed by recording the number of bees of each treatment that entered the observation hive through the ramp. Bees were classified as carrying or not carrying pollen on their pollen baskets. The proportion of bees participating in each foraging task (foraging trips with or without pollen) for each of the treatments, was calculated. This calculation was performed by dividing the number of forager bees with or without pollen over the number of bees remaining alive for each treatment per observation day.

After four weeks, when both hygienic and foraging assessments were completed, the remaining live marked bees were collected from the observation hives and frozen. Then, 30 randomly chosen bees from each treatment were sampled and *N. ceranae* infection intensity was determined by counting spores as described previously, to ensure that infections had developed in the experimental bees.

### 2.9. Proboscis Extension Reflex (PER)

Memory retention was assessed with the PER assay by individually feeding newly emerged bees with the treatments as described previously, then transferring 80 into a screened hoarding cage (10 × 15 × 15 cm). Bees were provided with sterile dH_2_O and 50% sucrose syrup *ad libitum* and were kept in an incubator at 32 ℃ and 60% RH for up to 20 days.

The PER test was performed with 13- to 19-day-old bees following standard protocols [33]. On the first day of evaluation, each bee was manually removed from its cage, and transferred to a glass vial (1 × 4 cm), which was then capped. The vial was placed on ice for 3 min to reduce the bee’s motion to facilitate its manipulation. Then, to immobilize the bee, its abdomen was introduced into a plastic tube (6 × 3 cm) of a stationery marker and wrapped with Parafilm (American National Can, Neenah, WI, USA) around its thorax; the head of the bee was free to move. Groups of 10 to 15 bees held in tubes placed in a plastic rack in an up-standing position were permitted to rest during 24 h before performing the PER test, and were fed three times per day with 10 µL of 50% sucrose syrup.

Experimental bees were trained to memorize the odor of clove oil (Sigma). A 30-mL syringe containing a piece of filter paper (Whatman No. 1) soaked with 20 µL of clove oil was used to project scented air to the bee held in the plastic tube at 5 cm from the syringe tip. The syringe plunger was slowly pushed to project the clove oil scent towards the bee. To make the scent flow, an aluminum foil ventilation pipe (10 × 100 cm) was placed about 1 cm behind the immobilized bee and a fan (Hawaiian Breeze, Canadian Tire, Guelph, ON, Canada) was installed with its rear end facing the opposite edge of the pipe so that the air was exhausted out of the testing arena. PER involved training the bees three times, followed by three PER measurements and ending with a sugar response (SR) evaluation. Training was done as per Denker et al. [34]. PER measurements were performed at 2, 24, and 48 h after the last training to evaluate short, medium, and long-term memory, respectively [33]. Tests comprised only the exposure to the scent for 5 s without touching the bee’s antennae and without feeding. If the bee extended its proboscis, the event was recorded as positive. Bees were fed daily 2 h before or after the tests. Immediately after the 48 h PER measurement, a SR evaluation was done by touching the antenna of the bee with a toothpick containing a small drop of 50% sucrose syrup. Results for bees with no ability to extend the proboscis during the SR evaluation were discarded from the analyses. A minimum of 50 bees were required to pass the SR evaluation for each of the treatments. Three replications were performed.

### 2.10. Statistical Analyses

Percentage or proportion data from survivorship, hygienic behavior and foraging behavior were arcsine square-root transformed to normalize their distributions before analyzing them. The transformed data were then subjected to two-way analyses of variance (ANOVA). *N. ceranae* spore counts data were square-root transformed before being analyzed by repeated-measures ANOVA. Means were compared with Fisher Protected LSD tests when significance was detected. The proportion of surviving bees at 30 dpt in the observation hives as well as the PER data were subjected to contingency table analysis with Chi square tests. These analyses were performed using R 3.3.1 (R Development Core Team, Auckland, New Zealand).

## 3. Results

### 3.1. N. ceranae Infection Intensity 

The numbers of *N. ceranae* spores/bee in non-inoculated control bees were zero at both 10 and 20 dpt, whereas the *N. ceranae* + no treatment bees had 2.20 × 10^6^ spores/bee at 10 dpt increasing to 18.67 × 10^6^ spores/bee at 20 dpt (Table 1). There were significant differences in number of *N. ceranae* spores/bee, with *N. ceranae* + chitosan treated bees having the lowest spore counts at 10 dpt (F_5, 12_ = 3.85, *p* < 0.05). At 20 dpt, the *N. ceranae* + chitosan, the *N. ceranae* + peptidoglycan and the *N. ceranae* + zymosan treated bees, in that order, had significantly lower *N. ceranae* spores/bee relative to the *N. ceranae* + no treatment and *N. ceranae* + flagellin treated bees (F_5, 12_ = 10.64, *p* < 0.001). Thus, chitosan and peptidoglycan were selected for further testing. 

### 3.2. Bee Survivorship 

**Figure 1:**
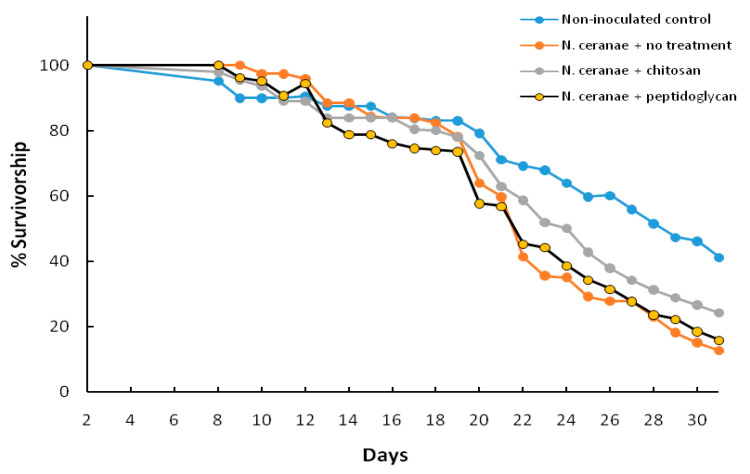
Percentage of bees that survived during 31 days in observation hives after the bees were inoculated with 1 × 10^5^ spores of *N. ceranae* only (*N. ceranae* + no treatment), inoculated with *N. ceranae* and 1.0 µg/µL of chitosan (*N. ceranae* + chitosan), *N. ceranae* and 1.0 µg/µL of peptidoglycan (*N. ceranae* + peptidoglycan) or not inoculated or treated (Non-inoculated control) (n = 500 per treatment).

Significant effects for treatment and age were detected when the proportions of surviving bees at the sampled days were analyzed (F_3, 200_ = 18.80, *p* < 0.0001 and F_24, 200_ = 44.31, *p* < 0.0001, respectively; Figure 1). No noticeable differences in the proportion of surviving bees between the treatments was evident up to 21 dpt, but a higher proportion of the negative control bees remained alive between 22 and 31 dpt followed by *N. ceranae* + chitosan, *N. ceranae* + peptidoglycan and *N. ceranae* + no treatment. By 31 dpt, the proportion of live bees for non-inoculated control, *N. ceranae* + chitosan, *N. ceranae* + peptidoglycan and *N. ceranae* + no treatment, was 0.41, 0.22, 0.16, and 0.12, respectively, which were each significantly different from each other (*p* < 0.05 based on χ^2^ tests). Thus, while chitosan and peptidoglycan treatments both reduced spore counts to levels not significantly different from each other (Table 1), chitosan gave a greater improvement to survivorship (Figure 1). 

### 3.3. Hygienic Behavior

**Figure 2:**
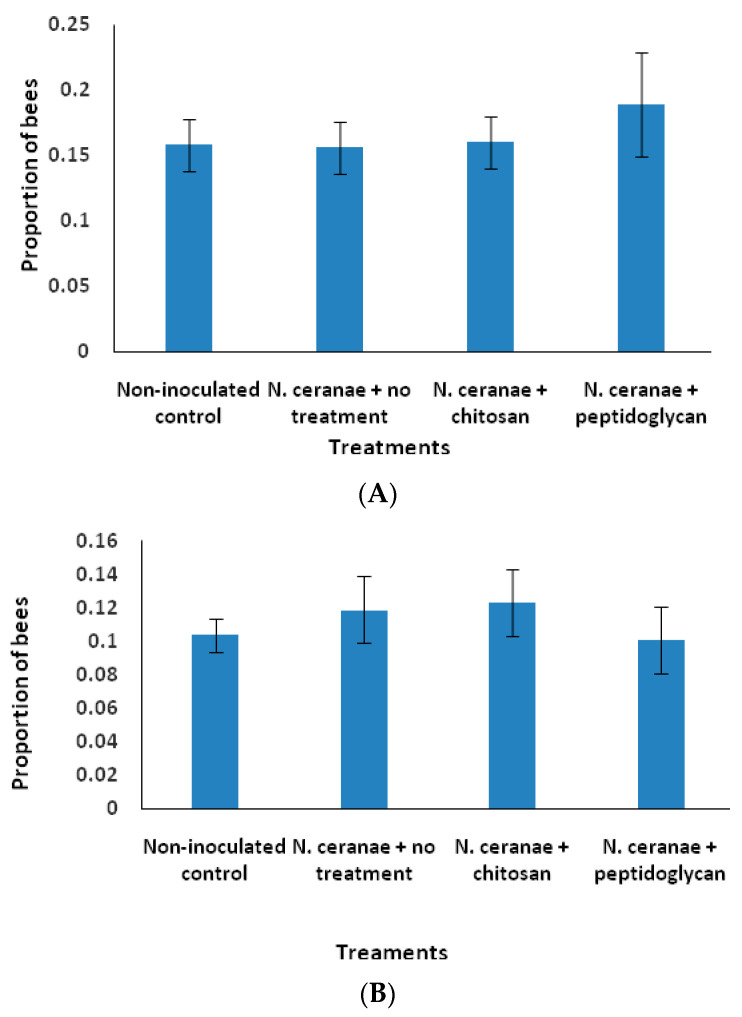
(**A**) Proportion (± SE) of worker honey bees that uncapped cells from a comb section containing freeze-killed brood. (**B**) Proportion (± SE) of worker honey bees that removed dead pupae from a comb section containing freeze-killed brood. Bees were inoculated with 1 × 10^5^ spores of *N. ceranae* only (*N. ceranae* + no treatment), inoculated with *N. ceranae* and 1.0 µg/µL of chitosan (*N. ceranae* + chitosan), *N. ceranae* and 1.0 µg/µL of peptidoglycan (*N. ceranae* + peptidoglycan) or not inoculated or treated (Non-inoculated control) (n = 500 per treatment).

No differences between non-inoculated control, *N. ceranae* + no treatment, chitosan or peptidoglycan treatments were found for the proportion of bees conducting cell uncapping (F_3, 32_ = 0.23, *p* > 0.05; Figure 2A) or brood removal (F_3, 32_ = 0.45, *p* > 0.05; Figure 2B). However, there were effects of colony with bees in colony one uncapping and removing significantly more brood than bees in the other two colonies (F_2, 33_ = 70.38, *p* < 0.0001, F_2, 33_ = 26.74, *p* < 0.0001, respectively). No treatment × colony interactions were detected for any of the two hygienic behavior variables (*p* > 0.05). 

### 3.4. Foraging Behavior

**Figure 3:**
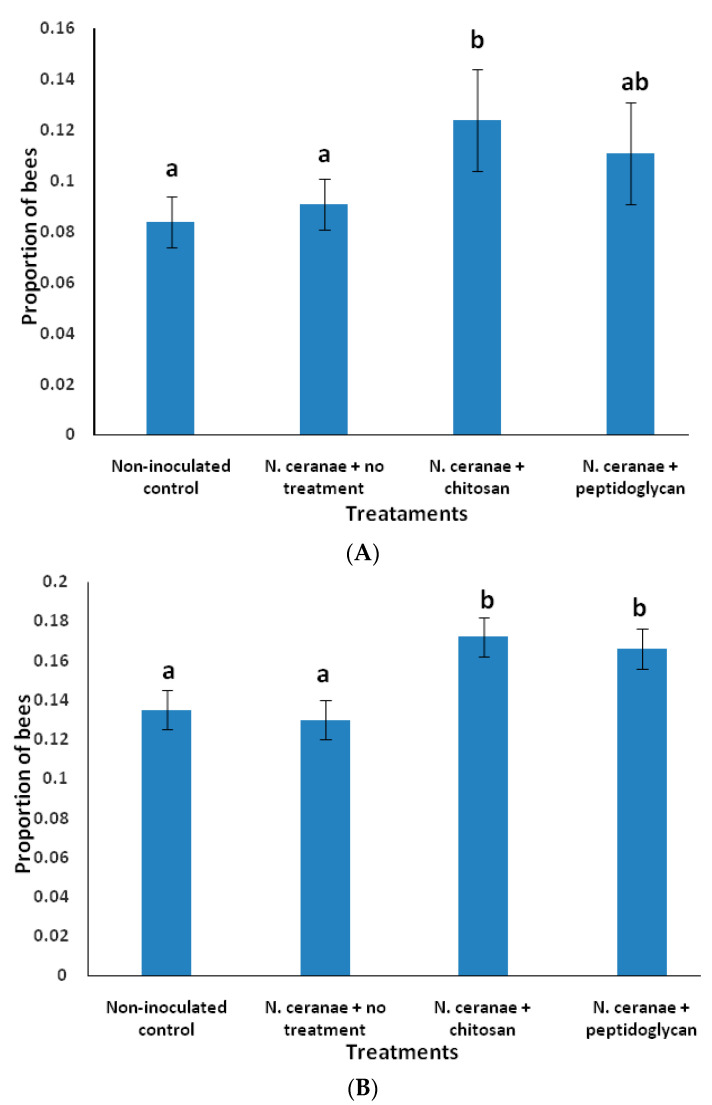
(**A**) Proportion (±SE) of worker honey bees that performed pollen foraging trips in 4 h of observation per day. (**B**) Proportion (±SE) of worker honey bees that performed non-pollen foraging trips (presumably nectar trips) in 4 h of observation per day. Bees were inoculated with 1 × 10^5^ spores of *N. ceranae* only (*N. ceranae* + no treatment), inoculated with *N. ceranae* and 1.0 µg/µL of chitosan (*N. ceranae* + chitosan), *N. ceranae* and 1.0 µg/µL of peptidoglycan (*N. ceranae* + peptidoglycan) or not inoculated or treated (Non-inoculated control) (n = 500 per treatment). Different literals above bars indicate significant differences based on ANOVA and Fisher protected LSD tests performed on arcsine square-root transformed data. Actual non-transformed values are depicted.

For the proportion of bees that performed pollen foraging trips, there was a significant increase with the *N. ceranae* + chitosan treatment compared to the non-inoculated control, the *N. ceranae* + no treatment and the *N. ceranae* + peptidoglycan treatment (F_3, 192_ = 3.82, *p* = 0.0110), which were not significantly different from each other (Figure 3A). There were also significant effects for day of observation (F_7, 192_ = 13.08, *p* < 0.0001), colony (F_2, 192_ = 106.41, *p* < 0.0001), and day × colony interaction (F_14, 192_ = 12.93, *p* < 0.0001) (Figure 3A).

For the proportion of bees that performed non-pollen foraging trips (presumably nectar trips), there was a significant increase with the *N. ceranae* + chitosan and *N. ceranae* + peptidoglycan treatments compared to the non-inoculated control and *N. ceranae* + no treatment (F_3, 192_ = 6.68, *p* = 0.0003), which were not significantly different from each other (Figure 3B). There was also a significant effect for day of observation (F_7, 192_ = 14.69, *p* < 0.0001), colony (F_2, 192_ = 7.28, *p* < 0.001), and day x colony interaction (F_14, 192_ = 19.71, *p* < 0.0001).

### 3.5. Memory Retention

The only differences between treatments for the percentage of bees that responded positively during the PER assay was for a lower percentage for the *N. ceranae* + chitosan treatment at 2 and 24 h (Table 2). However, at 48 h, no differences were detected for any of the treatments.

## 4. Discussion

This study showed that chitosan and peptidoglycan provide benefits by partially reducing *N. ceranae* spore numbers while increasing survivorship compared to *N. ceranae* infected bees. Also, chitosan and peptidoglycan improved aspects of foraging behavior even more than in healthy bees, showing that they may act as stimulators of important honey bee behaviors. 

*N. ceranae*-infected bees lived significantly less time than non-infected bees. Decreased survival of bees caused by *N. ceranae* infection in this study agrees with several other studies conducted with individual bees in cages [4,35,36,37] or colonies of bees [38,39,40]. Bees infected with *N. ceranae* live less time than non-infected bees probably because the fungus impairs their digestive functions and consequently affects nutrient absorption. Hence, infected bees would gain less energy from nutrients compared to healthy bees [36] and may eventually die of starvation and lack of energy. Furthermore, Mayack et al. [41] suggested that *N. ceranae* causes significant regulatory changes in the highly conserved octopamine hormone stress pathway, affecting the bee’s life span and possibly their age polyethism. This study showed that both chitosan and peptidoglycan reduced *N. ceranae* spore counts by more than 60%, which confirms a previous report on chitosan’s partial suppression of *N. ceranae* infections in honey bees [42]. However, although both PAMPs similarly reduced *N. ceranae* spore counts, chitosan was more effective in increasing survivorship of *N. ceranae* infected bees. Perhaps chitosan has other beneficial effects for the bees reflected in survivorship. For example, chitosan treatment of bees can reduce oxidative stress [43]. 

One effect of infection by *N. ceranae* appears to be the suppression of the immune system of honey bees. For example, expression of the immune related gene, *hymenoptaecin*, has been reported to be down-regulated at 3 and 6 dpi [44], 2 dpi [45] and 5 dpi [25]. The beneficial effects of 1.0 µg/µL chitosan and peptidoglycan on honey bees may be related to changes in gene expression. For bees inoculated with *N. ceranae*, expression of *hymenoptaecin* was significantly higher with peptidoglycan and chitosan than non-treated control bees at 5 dpt. Expression of *defensin 2* in bees inoculated with *N. ceranae* was significantly higher with peptidoglycan and chitosan compared to non-treated control bees by 1 and 2 dpt, respectively. For *blue cheese*, significantly higher expression was detected in *N. ceranae* infected bees by 1 dpt with chitosan and 10 dpt with peptidoglycan compared to infected non-treated bees. Thus, one effect of these treatments may be to reverse the suppression of the honey bee immune system by *N. ceranae*. 

This is the first study to evaluate the hygienic behavior of *N. ceranae* infected bees, as well as the effect of chitosan and peptidoglycan treatments on this behavior. Hygienic behavior provides resistance to honey bees against several brood diseases and to the parasitic mite *V. destructor* [46,47,48,49]. Thus, any pathogen that could impair the ability of honey bees to defend themselves against brood diseases and parasites would have a negative effect on honey bee colonies. However, no significant effect of *N. ceranae* infections or chitosan and peptidoglycan treatments on the two variables of hygienic behavior analyzed in this study was detected. By the age when the bees were evaluated for hygienic behavior, the impact of *N. ceranae* infections may not have yet been significant on bee health as it takes at least two weeks for spore counts to reach high levels [50]. Thus, testing older bees might have shown an effect. It was noteworthy that neither chitosan nor peptidoglycan had any negative effect on the hygienic behavior of the bees. Thus, their use would not affect this important behavior. 

For foraging behavior, *N. ceranae* infection did not affect either the proportion of bees that performed pollen or non-pollen foraging trips. Conversely, Kralj and Fuchs [10] reported a reduced number of foraging trips for *N. ceranae* infected bees compared to non-infected bees in one of their experiments, which was believed to be due to impaired orientation skills of *N. ceranae*- and *N. apis*-infected foragers compared to healthy bees. However, their results were inconsistent because in a second identical experiment, they did not find differences in foraging trips between infected and non-infected bees. In another study, Alaux et al. [39] found that *Nosema* infections did not reduce foraging behavior in young bees, but it was reduced in 20-day-old bees or older. One difference between the studies is that this study calculated foraging relative to the number of bees that were alive at observation times, whereas Alaux et al. [39] and Kralj and Fuchs [10] counted the number of live bees relative to the starting number of bees. Thus, studies using older bees may have reported differences in foraging activity because the number of infected bees at observation times was lower than that of non-inoculated bees. Another factor that may affect the impact of *N. ceranae* infection on foraging behavior is the inoculum dose. Odemer et al. [51] found that infection increased foraging behavior, but they fed a lower dose (1.4 × 10^4^ spores of *N. ceranae* per bee) than the 1.0 × 10^5^ spores per bee used in this study, as well as Alaux et al. [39] who used 2.0 × 10^4^ spores per bee and Kralj and Fuchs [10] who used 1.2 × 10^5^ spores with bulk feeding. Thus, a number of factors, such as bee age and pathogen dose, could be responsible for the differences in results. This emphasizes the need for standardized conditions to be able to compare studies of how *Nosema* infections affect the foraging behavior of honey bees.

This study is the first to find a positive effect of two natural compounds on a behavior of honey bees, indicating that they can have effects independent of their ability to restrain the proliferation of *Nosema* spores. Chitosan treatment increased both the number of pollen and non-pollen foraging trips, while peptidoglycan significantly increased only non-pollen foraging trips. Increased foraging activity due to chitosan and peptidoglycan might be beneficial because colonies would have more food stores for winter survival and honey production. The number of foraging trips has been shown to be directly related to honey production and overwintering survival of honey bee colonies [30,52]. However, further investigation is warranted to evaluate whether chitosan and peptidoglycan treatments can affect honey production and overwintering survival. Future studies should also test the compounds without *N. ceranae* infection to determine if they would also increase foraging. 

The results from the PER assays in this work are consistent with those of two previous studies showing no effects of *N. apis* or *N. ceranae* infections on honey bee learning and memory using the PER assay [16,53]. Conversely, Gage et al. [54] showed that *N. ceranae* negatively impacts PER in honey bees. While Gage et al. [54] used the same level of inoculum, they tested 7- to 15-day-old bees versus 13- to 19-day-old bees in this study. Thus, the age of the bees could be having an impact on the results, warranting further investigation. 

While there were no differences at 48 h, chitosan had a negative effect on PER at 2 and 24 h post training. In contrast, peptidoglycan never significantly affected bee memory. The age of the bees first evaluated by the PER assay was similar to that of bees assessed for foraging behavior during the first days of the observation trials. Thus, the negative effects of chitosan versus the lack of an effect of peptidoglycan on bee memory did not appear to be reflected in chitosan and peptidoglycan both improving aspects of foraging. Even though there is a correlation between learning ability and foraging behavior of honey bees [55], it may be that any impairment of learning by chitosan measured with the PER assay in this study was not sufficient to negatively affect aspects of learning related to foraging after chitosan treatment. One possibility is that chitosan negatively affected PER in the short term because chitosan can be a central nervous system depressant, slowing down brain activity in vertebrates [56]. Whether there are any long-term effects of that on behaviors related to learning remains to be investigated.

While PAMPs are widely studied for their effects on infections, this study demonstrates that they can also affect the learning ability and memory retention of bees as well as foraging behaviors of honey bees. Considering the importance of those functions for honey bees, it is key to study such compounds for the benefit of honey bees aside for their potential to control *N. ceranae*. While the level of control of the pathogen was limited, further work on these and other natural compounds, combinations, doses, formulations, and other factors may be able to increase the level of *N. ceranae* control. Examples of other compounds that could be tested include β–glucans, which are immune-modulator molecules that have been shown to reduce viral loads and increase survival rate of honey bees parasitized with *Varroa destructor* [57]. Natural compounds also have the benefit of reducing the risks of antibiotic honey contamination and development of antibiotic resistance. 

## 5. Conclusions

This study showed that chitosan can reduce *N. ceranae* spore multiplication, improve survivorship of infected bees, and increase both pollen and non-pollen foraging without adversely affecting hygienic behavior. However, chitosan caused a significant decrease in memory retention for the first 24 h of the PER tests, although at 48 h no effect was detected. By comparison, peptidoglycan is also promising in that it achieved similar results, except for not improving survivorship of *N. ceranae*-infected bees and not increasing pollen foraging. However, peptidoglycan had the advantage of not showing a negative effect on memory retention. Although successful, the reduction in spore numbers with either compound is limited. A question is whether these compounds can be made to work better, such as by providing them continuously, rather than only one initial feeding, and testing at a colony level, where conditions are more difficult to control.

## Figures and Tables

**Table 1:** Mean (±SE) *N. ceranae* spores per bee at 10 and 20 dpt following no inoculation (non-inoculated control) or inoculation with 1 × 10^5^ spores of *N. ceranae* only (*N. ceranae* + no treatment), *N. ceranae* and 1.0 µg/µL chitosan (*N. ceranae* + chitosan), 0.10 µg/µL flagellin (*N. ceranae* + flagellin), 0.125 µg/µL zymosan (*N. ceranae* + zymosan) or 1.0 µg/µL peptidoglycan (*N. ceranae* + peptidoglycan).

Treatment	10 dpt Spores/Bee (±SE)	20 dpt Spores/Bee (±SE)
Non-inoculated control	0 (0)	0 (0)
*N. ceranae* + no treatment	2.20 × 10^6 ab^ (9.8 × 10^5^)	18.67 × 10^6 a^ (3.8 × 10^6^)
*N. ceranae* + chitosan	8.17 × 10^5 b^ (5.9 × 10^5^)	6.63 × 10^6 c^ (2.4 × 10^6^)
*N. ceranae* + flagellin	3.22 × 10^6 a^ (9.2 × 10^5^)	15.88 × 10^6 ab^ (2.9 × 10^6^)
*N. ceranae* + zymosan	1.73 × 10^6 a^ (1.1 × 10^6^)	7.97 × 10^6 bc^ (4.1 × 10^6^)
*N. ceranae* + peptidoglycan	1.18 × 10^6 b^ (8.1 × 10^5^)	6.58 × 10^6 c^ (2.3 × 10^6^)

Different literals indicate significant differences based on ANOVA and Fisher’s LSD tests on transformed data. n = 180 bees per treatment.

**Table 2:** Percentage of bees that showed positive proboscis extension reflex (PER) at 2, 24, and 48 h. The bees were either non-inoculated with *N. ceranae*, inoculated with 1 × 10^5^ spores of *N. ceranae* only (*N. ceranae* + no treatment), *N. ceranae* and 1.0 µg/µL chitosan (*N. ceranae* + chitosan), and 1.0 µg/µL peptidoglycan (*N. ceranae* + peptidoglycan) (n = 50 per treatment).

Treatment	2 h	24 h	48 h
Non-inoculated control	50 ^a^	28 ^a^	20 ^a^
*N. ceranae* + no treatment	32 ^a^	24 ^ab^	24 ^a^
*N. ceranae* + chitosan	8 ^b^	12 ^b^	18 ^a^
*N. ceranae* + peptidoglycan	34 ^a^	26 ^ab^	24 ^a^

Different literals indicate significant differences based on contingency table analyses and χ^2^ tests.
